# Association between APOE-ε4 allele and cognitive function is mediated by Alzheimer’s disease pathology: a population-based autopsy study in an admixed sample

**DOI:** 10.1186/s40478-023-01681-z

**Published:** 2023-12-19

**Authors:** Regina Silva Paradela, Alberto Fernando Oliveira Justo, Vítor Ribeiro Paes, Renata E. P. Leite, Carlos A. Pasqualucci, Lea T. Grinberg, Michel Satya Naslavsky, Mayana Zatz, Ricardo Nitrini, Wilson Jacob-Filho, Claudia Kimie Suemoto

**Affiliations:** 1 Division of Geriatrics, University of São Paulo Medical School, 455 Doutor Arnaldo Avenue, room 1355, São Paulo, SP Brazil; 2 Department of Pathology, University of Sao Paulo Medical School, São Paulo, Brazil; 3 Memory and Aging Center, University of California, San Francisco, USA; 4 Human Genome and Stem Cell Center, Biosciences Institute, University of São Paulo, São Paulo, Brazil; 5 Department of Neurology, University of São Paulo Medical School, São Paulo, Brazil

**Keywords:** Apolipoprotein E, Cognition, Dementia, Neuritic plaques, Neurofibrillary tangles, Mediation analysis

## Abstract

**Background:**

Apolipoprotein E ε4 allele (APOE-ε4) is the main genetic risk factor for late-onset Alzheimer’s disease (AD) and may impact cognitive function also via other neuropathological lesions. However, there is limited evidence available from diverse populations, as APOE associations with dementia seem to differ by race. Therefore, we aimed to evaluate the pathways linking APOE-ε4 to cognitive abilities through AD and non-AD neuropathology in an autopsy study with an admixed sample.

**Methods:**

Neuropathological lesions were evaluated following international criteria using immunohistochemistry. Participants were classified into APOE-ε4 carriers (at least one ε4 allele) and non-carriers. Cognitive abilities were evaluated by the Clinical Dementia Rating Scale sum of boxes. Mediation analyses were conducted to assess the indirect association of APOE-ε4 with cognition through AD-pathology, lacunar infarcts, hyaline arteriosclerosis, cerebral amyloid angiopathy (CAA), Lewy body disease (LBD), and TAR DNA-binding protein 43 (TDP-43).

**Results:**

We included 648 participants (mean age 75 ± 12 years old, mean education 4.4 ± 3.7 years, 52% women, 69% White, and 28% APOE-ε4 carriers). The association between APOE-ε4 and cognitive abilities was mediated by neurofibrillary tangles (β = 0.88, 95% CI = 0.45; 1.38, *p* < 0.001) and neuritic plaques (β = 1.36, 95% CI = 0.86; 1.96, *p* < 0.001). Lacunar infarcts, hyaline arteriosclerosis, CAA, LBD, and TDP-43 were not mediators in the pathway from APOE-ε4 to cognition.

**Conclusion:**

The association between APOE-ε4 and cognitive abilities was partially mediated by AD-pathology. On the other hand, cerebrovascular lesions and other neurodegenerative diseases did not mediate the association between APOE-ε4 and cognition.

**Supplementary Information:**

The online version contains supplementary material available at 10.1186/s40478-023-01681-z.

## Introduction

The apolipoprotein E gene ε4 allele (APOE-ε4) is the main genetic risk factor for late-onset Alzheimer’s disease (AD) [1]. Compared to the APOE-ε3/ε3 genotype, having at least one APOE-ε4 allele increases the risk of cognitive decline and dementia [2–4]. According to previous evidence, the association of APOE-ε4 with cognition and dementia seems to be mediated by an increased burden of AD-pathology: neurofibrillary tangles (NFT) formed by hyperphosphorylated tau proteins and neuritic plaques (NP) composed of β-amyloid peptides [5–7]. However, the APOE alleles are also associated with a higher risk of cerebrovascular lesions, including atherosclerosis, brain infarcts, hyaline arteriosclerosis, and cerebral amyloid angiopathy (CAA) [8–10]. In addition, APOE is related to non-AD neuropathological alterations, such as Lewy body dementia (LBD) characterized by aggregates of the α-synuclein protein and limbic-predominant age-related TAR DNA-binding protein 43 (TDP-43) encephalopathy [10–15]. Thus, these lesions could mediate the APOE association with cognitive function.

Few population-based studies have investigated these pathways through cerebrovascular lesions and non-AD neuropathology using causal mediation approaches, which estimate direct and indirect effects considering the interactions between the exposure and the mediator [16]. APOE-ε4 was related to an increased risk of hippocampal sclerosis mediated by the burden of TDP-43 pathology in an autopsy study with 1042 older adults [17]. In 17,244 participants from five longitudinal studies, the APOE-ε4 allele was associated with a higher AD risk through their indirect effects on increasing body mass index (BMI) [18]. Recently, it was demonstrated that the association of APOE-ε4 with lower late-life global cognitive scores was mediated by a higher burden of AD-pathology, as well as the presence of CAA, LBD, and TDP-43 in 1,671 participants, while cerebrovascular lesions were not mediators of the association between APOE alleles and cognitive function  [19].

Most studies so far included predominantly White participants with high educational attainment. However, studies suggested that the association between APOE-ε4 and cognition may differ by race, highlighting the need to understand the pathways through which APOE influences cognitive function in diverse populations [3, 4]. Therefore, we aimed to evaluate the direct and indirect effects of neurodegenerative and cerebrovascular lesions on the association between APOE alleles and cognitive abilities by using data from a community-based sample of ethnically diverse participants.

## Methods

### Participants

We used data from the Biobank for Aging Studies of the University of Sao Paulo Medical School [20]. In Sao Paulo, individuals who die from a non-traumatic death of an unknown cause are submitted to an autopsy exam. Autopsies were performed at the Sao Paulo Autopsy Service, and the brains were donated by the deceased’s next of kin (NOK) [20, 21]. The NOK signed an informed consent allowing the brain collection and provided sociodemographic and clinical information about the deceased. The local ethics committee approved this study.

We included individuals aged 50 years or older at the time of death whose NOK had at least weekly contact with the deceased in the six months before death and with complete APOE data (n = 1151). Then, we excluded individuals with missing data for neuropathological variables (n = 482) and for sociodemographic and clinical information (n = 21). We analyzed data from 648 participants (Additional file 1: Fig. S1).

### Neuropathological assessment

Brains were obtained within 24 h after death (mean post-mortem interval of 14.2 ± 4.8 h), and the brainstem and cerebellum were removed before further processing, after which the brain was separated into two hemispheres. Selected areas were collected from the right hemisphere and frozen at − 80 °C for future studies. The brainstem, left hemisphere of the brain, and the cerebellum were fixed in 4% buffered paraformaldehyde. The fixation time is 15 days, but in exceptional cases, this time can range from 15 to 45 days, based on the quality assessment of the brain fixation after 15 days by our neuropathologists. Then, the fixed hemisphere was sectioned into the following areas: middle frontal gyrus, middle and superior temporal gyri, angular gyrus, superior frontal and anterior cingulate gyrus, visual cortex, hippocampal formation at the level of the lateral geniculate body, amygdala, basal ganglia at the level of the anterior commissure, thalamus, midbrain, pons, medulla oblongata, and cerebellum [21]. All brain regions were embedded in paraffin, sectioned into 5-μm-thickness sections, and stained with hematoxylin and eosin. To analyze the neuropathological lesions, we performed immunohistochemistry with antibodies against β-amyloid (4G8, 1:10.000; BioLegend #800,701), phosphorylated tau (AT8, 1:400; Invitrogen MN1020), TDP-43 (1:500, BioLegend #829,901), and α-synuclein (LB509, 1:500; Sigma-Aldrich MABN824) in the selected brain regions (Additional file 1: Table S1)  [20, 21].

AD-pathology was scored using the Braak and Braak staging system for NFT and the CERAD criteria for NP. The Braak and Braak staging is a system to measure the progression of NFT into six stages. The NFT changes start in the transentorhinal regions (stage I) and spread to the cornu ammonis (CA) 1 of the hippocampus (stage II). As the pathology progresses, the lesions extend to the subiculum, basal ganglia, and amygdala (stage III); and accumulate in the isocortex, mainly the temporal region (stage IV). Then, the lesions extend widely into the whole CA region, and the thalamus and hypothalamus are committed (stage V). Finally, the pathology reaches the occipital lobe and the fascia dentata of the hippocampus (stage VI) [22, 23]. According to these stages, NFT accumulation was categorized into 0-II, III-IV, or V-VI [24]. CERAD criterion is a semiquantitative approach that categorizes the frequency of neocortical NP into the following stages: none (0), sparse (I), moderate (II), and frequent (III) [25]. We categorized these frequencies into none or sparse, moderate, and frequent.

LBD was classified according to the presence of α-synuclein using the Braak staging for Parkinson's disease (PD) [26]. Therefore, we used the term LBD for all diseases related to Lewy bodies, such as PD, PD dementia, and Lewy body dementia. The diagnosis of LBD was considered when the Braak staging for PD ≥ III [21, 27]. The presence of TDP-43 was determined using immunostaining to detect TDP-43 in the hippocampal formation and amygdala.

Cerebrovascular lesions were evaluated microscopically using hematoxylin and eosin-stained slides in all sampled areas. Lacunar infarcts were registered by topography, stage, size, and number. The presence of lacunar infarcts was defined as one or more infarcts measuring ≤ 1.5 cm in any of the regions of the brain described above. Hyaline arteriosclerosis was classified according to the presence of moderate and/or severe microvascular changes in three or more cortical regions. The evaluation of CAA was made according to the localization, as well as the severity and presence of capillary amyloid deposition. CAA was classified as present when it was widespread in the parenchyma in at least three different cortical areas [21].

### Cognitive assessment

Cognitive abilities were evaluated using the Clinical Dementia Rating (CDR) [28]. CDR is a semistructured interview that is applied to the informant and the participant. However, we used only the informant section of the CDR. The NOK, who had at least weekly contact with the deceased during the six months prior to death, answered questions regarding the cognitive abilities of the deceased three months before death to avoid the cognitive changes that are usually present next to the moment of death. Cognitive abilities were assessed in six domains (memory, orientation, judgment and problem-solving, community affairs, home, hobbies, and personal care). Each domain is scored on a five-point scale used to rate dementia severity: normal cognition (CDR = 0); cognitive impairment (CDR = 0.5); mild dementia (CDR = 1); moderate dementia (CDR = 2); and severe dementia (CDR = 3). The global CDR score is obtained through an algorithm [28]. We also computed the CDR sum of boxes (CDR-SOB), which is the sum of the scores in each domain that ranges from 0 to 18, with higher scores indicating worse cognitive abilities [29].

### APOE Genotyping

DNA samples were obtained from blood or brain tissue. The APOE polymorphism was genotyped using either Illumina OmniExpress 700 k microarray or Illumina BeadXpress custom genotyping panel. APOE common alleles (ε2, ε3, and ε4) were genotyped directly by real-time polymerase chain reaction (PCR) using allele-specific amplification or after imputation of rs429358 to compose haplotypes [30].

### Other variables

Other study variables were age at death, sex, education, and race, categorized as White, Black, Brown (admixed of Black and White), and Asian. Black and Brown participants were analyzed as Black. We also collected information on previous diagnoses of diabetes, hypertension, dyslipidemia, and heart disease (cardiac failure and/or coronary artery disease). The NOK reported all the sociodemographic and clinical variables except BMI, which was calculated using the measured height and weight before the autopsy exam. Physical activity was defined as present when the NOK reported that the deceased had regularly engaged in physical activities, such as household chores, walking, and practicing physical exercises. Smoking status and alcohol use were also reported by the NOK and classified as never, current, or former use. Age and sex were obtained from government-issued documents.

### Statistical analysis

Descriptive statistics were presented as mean and standard deviation (SD) for quantitative variables and relative frequencies for categorical variables. Participants were categorized into APOE-ε4 carriers and non-carriers according to the presence of at least one ε4 allele. Then, we compared the sociodemographic and clinical characteristics of the two groups (APOE-ε4 carriers vs. non-carriers) using the unpaired t-test for quantitative variables and the Chi-squared or Fisher's exact test for categorical variables.

Estimates of the mediation models were calculated based on the product-of-coefficients method [16]. According to this approach, the direct effect represents the difference in the outcome when the exposure changes (APOE-ε4 carriers vs. non-carriers) and the mediator is held constant. The indirect effect represents the expected difference in the outcome when the mediator changes in the function of the values of the APOE categories (APOE-ε4 carriers vs. non-carriers) while the exposure (APOE categories) is held constant. In addition, the mediation model also estimated the total effect and the mediated proportion. The total effect is the effect of the APOE-ε4 on cognitive abilities, summing the direct and indirect effects. Mediated proportion represents the proportion of the total effect that is due to the APOE allele's effect on cognitive abilities through the mediators. [16, 31]

**Fig. 1:**
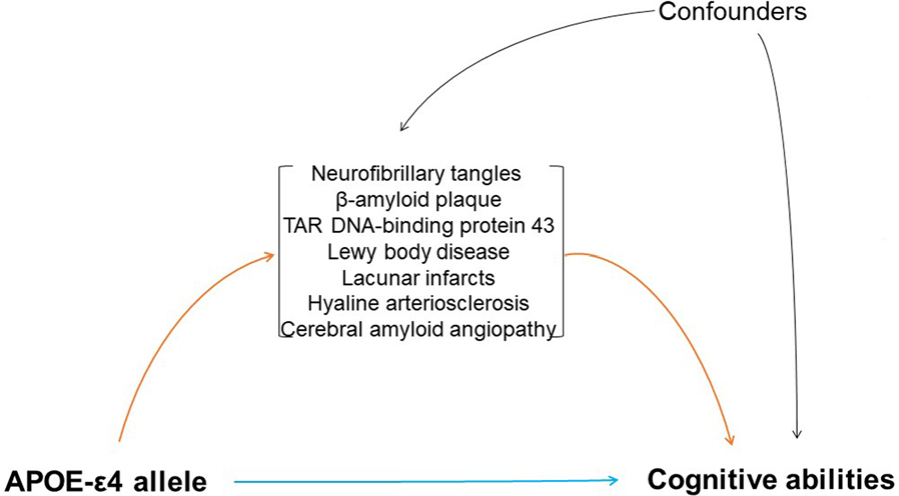
Directed acyclic graph showing the conceptual causal mediation model of the associations between the apolipoprotein E gene ε4 allele (APOE-ε4) and cognitive abilities decomposed into a direct (blue line) and indirect pathway (red line). Confounder variables were age, sex, race, education, hypertension, diabetes, dyslipidemia, heart disease, body mass index, alcohol use, smoking, and physical activity

The causal mediation analysis was conducted using the ‘mediate’ function of the R package mediation [32]. First, we fitted the mediator and outcome models separately. In the mediator models, each of the mediators (lacunar infarcts, hyaline arteriosclerosis, CAA, TDP-43, LBD, Braak score, and CERAD score) was entered as the outcome variables, and the APOE categories as the predictor in a linear regression model adjusted for sociodemographic (age, sex, race, and education) and clinical factors (hypertension, diabetes, dyslipidemia, heart disease, BMI, alcohol, smoking, and physical activity). In the outcome model, the CDR-SOB was the outcome variable, and the APOE categories and the mediators were predictors in a linear regression model adjusted for the set of confounders described above. Then, we entered these two models as arguments of the mediate function that decomposed the associations between APOE alleles and CDR-SOB into direct and indirect paths represented by the average direct effects (ADE) and the average causal mediation effects (ACME), respectively (Fig. 1). We verified the assumptions of the linear regression models, including normal quantile–quantile (Q–Q) plots and histograms of the residuals. In addition, the mediation effects were estimated with the default quasi-Bayesian Monte Carlo method and bootstrapping with 1000 simulations. We considered an alpha level of 5% in two-tailed tests.

## Results

In 648 participants, the mean age was 74.7 ± 12.0 years old, the mean education was 4.4 ± 3.7 years, 52% were women, and 69% were White. Subjects with at least one APOE-ε4 allele (n = 182) had fewer years of education (p = 0.01) and were more frequently Black (*p* = 0.001) than the APOE-ε4 non-carriers (n = 466) (Table 1).

**Table 1:** Sociodemographic and clinical characteristics of the sample (n = 648)

Variable	Overall (n = 648)	APOE-ε4 non-carriers (n = 466)	APOE-ε4 carriers (n = 182)	*p*
Age (y), Mean ± SD*ª*	74.7 ± 12.0	74.5 ± 12.0	75.0 ± 11.5	0.62
Education (y), Mean ± SDª	4.4 ± 3.7	4.6 ± 3.8	3.7 ± 3.4	0.01
Sex (%)^*b*^				
Female	52.3	52.6	51.6	0.90
Race (%)^*c*^				0.001
White	68.5	72.1	59.3	
Black	28.7	24.7	39.0	
Asian	2.8	3.2	1.7	
Hypertension (%)^*b*^	65.1	63.9	68.1	0.36
Diabetes (%)^*b*^	28.9	30.3	25.3	0.25
Dyslipidemia (%)^*b*^	9.7	9.23	11.0	0.59
Heart disease (%)^*b*^	31.3	31.8	30.2	0.78
Stroke (%)^*b*^	14.2	14.4	13.7	0.93
BMI (kg/m^2^), Mean ± SD*ª*	23.2 ± 5.0	23.3 ± 5.1	23.1 ± 5.0	0.60
Physical inactivity (%)^*b*^	51.2	49.4	56.0	0.15
Smoking (%)^*b*^				0.76
Never	49.1	48.3	51.1	
Current	19.8	20.4	18.1	
Former	31.2	31.3	30.8	
Alcohol use (%)^*b*^				0.94
Never	59.1	58.8	59.9	
Current	23.0	23.0	23.1	
Former	17.9	18.2	17.0	
CDR ≥ 0.5 (%)^b^	30.7	26.4	41.8	< 0.001
Braak & Braak score (%)^b^				< 0.001
0-II	59.3	63.1	49.5	
III-IV	27.9	27.0	30.2	
V-VI	12.8	9.9	20.3	
CERAD score (%)^b^				< 0.001
None or Sparse	71.8	78.3	54.9	
Moderate	15.6	13.9	19.8	
Frequent	12.7	7.7	25.3	
TDP-43 (%)^b^*	12.3	11.6	14.3	0.67
Lewy body disease (%)^b^**	12.8	12.7	13.1	1.00
Lacunar Infarcts (%)^b^	11.6	10.3	14.8	0.14
Hyaline arteriosclerosis (%)^b^	21.1	21.9	19.2	0.52
Cerebral Amyloid Angiopathy (%)^b^	7.1	4.5	13.7	< 0.001

^a^Student's *t*-test. ^b^Chi-square test. ^c^Fisher’s exact test

*SD* Standard deviation, *CDR* Clinical dementia rating. *CERAD* Consortium to establish a registry for Alzheimer’s disease. *TDP-43* TAR DNA-binding protein 43. Missing data were excluded for analyses with TDP-43 (*n = 310) and Lewy body disease (**n = 617)

**Fig. 2:**
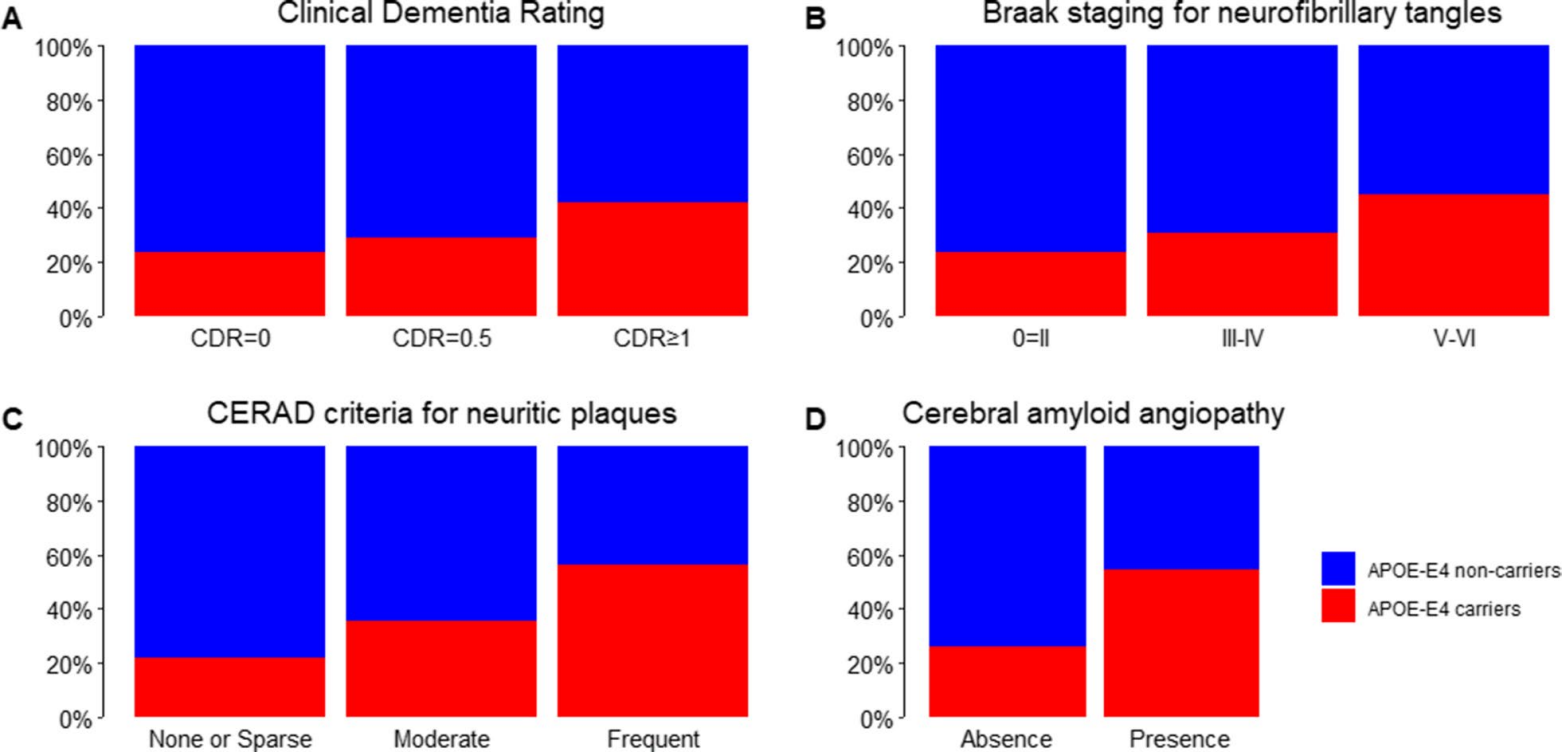
Associations of the Apolipoprotein E gene ε4 allele (APOE-ε4) with Clinical Dementia Rating (CDR) and neurodegenerative lesions. Compared to APOE ε4 non-carriers, APOE-ε4 carriers had **A** more cognitive impairment (CDR = 0.5) and probable dementia (CDR ≥ 1) (*p* < 0.001), **B** higher frequency of Braak staging ≥ III (*p* < 0.001), **C** moderate and frequent neuritic plaques (*p* < 0.001), and **D** cerebral amyloid angiopathy (*p* < 0.001). Chi-square tests were used for all comparisons

APOE-ε4 carriers had a higher frequency of cognitive impairment (CDR score ≥ 0.5) (Fig. 2A), higher scores of Braak staging for NFT (Fig. 2B) and CERAD score for NP (Fig. 2C), and a higher frequency of CAA (Fig. 2D) than APOE-ε4 non-carriers. However, no differences were found between the APOE groups for lacunar infarcts, hyaline arteriosclerosis, TDP-43, and LBD (Table 1).

### Neuropathological lesions as mediators of the association between apolipoprotein E gene and cognitive abilities

**Fig. 3:**
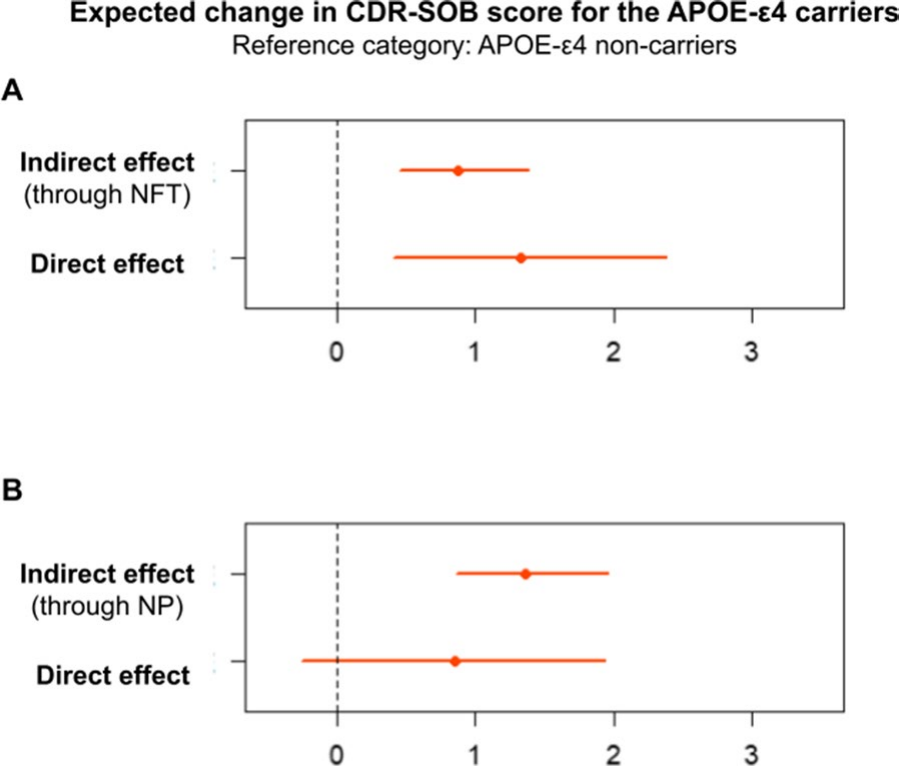
Estimates (points) and 95% confidence intervals for the direct and indirect effects through neurodegenerative lesions of the association between Apolipoprotein E gene ε4 allele (APOE-ε4) and Clinical Dementia Rating sum of boxes (CDR-SOB). **A** The Braak staging for neurofibrillary tangles (NFT) and **B** the Consortium to Establish a Registry for Alzheimer’s Disease (CERAD) criteria for neuritic plaques (NP) were significant mediators of the association between APOE-ε4 and cognitive abilities (*p* < 0.001). Direct paths remained significant in the model with the Braak score (*p* = 0.002) but were not significant when the CERAD score was entered as a mediator (*p* = 0.13)

Participants with the APOE-ε4 allele had higher CDR-SOB scores mediated by a higher burden of NFT (Braak score) and NP (CERAD score) in mediation analysis adjusted for age, sex, race, and education (Fig. 3, Panels A and B; Table 2). Forty percent and 62% of the association between APOE-ε4 and cognitive abilities were explained by the burden of NFT and NP measured respectively by Braak and CERAD scores. TDP-43, lacunar infarcts, hyaline arteriosclerosis, and CAA did not mediate the association between APOE-ε4 and cognitive abilities (Table 2). The direct pathway between APOE-ε4 and cognition remained significant in the presence of all mediators except TDP-43 (*p* = 0.07) and NP (*p* = 0.13), indicating that the APOE-ε4 allele can influence cognition through other paths independent of NFT, NP, TDP-43, lacunar infarcts, hyaline arteriosclerosis, and CAA (Table 2). Similar results were found in mediation analysis adjusted for sociodemographic and clinical factors (Additional file 1: Table S2).

**Table 2:** Decomposition of the total, direct, and indirect effects through neurodegenerative and cerebrovascular lesions of the association between Apolipoprotein E gene ε4 allele (APOE-ε4) and cognitive abilities (n = 648)

Outcome: Cognitive abilities measured by the clinical dementia rating sum of boxes								
	Total effect		Direct effect		Indirect effect		Proportion mediated	
	Estimate (95% CI)	*p*	Estimate (95% CI)	*p*	Estimate (95% CI)	*P*	Estimate (95% CI)	*p*
Neurodegenerative mediators								
Neurofibrillary tangles (n = 648)	2.21(1.20; 3.29)	< 0.001	1.33(0.42; 2.38)	0.002	0.88(0.45; 1.38)	< 0.001	0.40(0.21; 0.70)	< 0.001
Neuritic plaques (n = 648)	2.21(1.10; 3.24)	< 0.001	0.85( − 0.26; 1.94)	0.13	1.36(0.86; 1.96)	< 0.001	0.62(0.35; 1.22)	< 0.001
TDP-43 (n = 310)	1.35( − 0.04; 2.82)	0.06	1.25( − 0.13; 2.63)	0.07	0.10( − 0.16; 0.52)	0.50	0.07( − 0.35; 0.54)	0.52
Lewy bodies disease (n = 617)	2.04(0.97; 3.11)	< 0.001	2.03(1.00; 3.08)	< 0.001	0.01( − 0.12; 0.17)	0.92	0.003( − 0.07; 0.08)	0.92
Cerebrovascular mediators								
Lacunar infarcts (n = 648)	2.21(1.18; 3.31)	< 0.001	2.12(1.11; 3.23)	< 0.001	0.09( − 0.05; 0.26)	0.21	0.04( − 0.03; 0.13)	0.21
Hyaline arteriosclerosis (n = 648)	2.21(1.13; 3.24)	< 0.001	2.28(1.23; 3.33)	< 0.001	− 0.07( − 0.22; 0.05)	0.25	− 0.03( − 0.12; 0.02)	0.25
Cerebral Amyloid Angiopathy (n = 648)	2.21(1.13; 3.29)	< 0.001	2.08(0.93; 3.22)	< 0.001	0.12( − 0.07; 0.35)	0.20	0.06( − 0.03; 0.20)	0.20

Mediation analysis adjusted for age, sex, race, and education

APOE groups: Participants with at least one APOE-ε4 allele vs. APOE-ε4 non-carriers (reference group)

## Discussion

In an admixed sample, NFT and NP mediated, respectively, 40% and 62% of the association between the presence of at least one APOE-ε4 allele and poor cognitive abilities. Cerebrovascular lesions and non-AD neuropathologies were not significant mediators on the pathway linking APOE-ε4 to cognitive abilities.

Our results agree with prior studies that have found an association of APOE-ε4 with increased β-amyloid pathology, tau neurotoxicity, and cognitive impairment. However, the majority of these studies did not use mediation approaches and included non-diverse populations [7, 19, 33, 34]. Results from 272 Koreans who underwent PET scans showed that APOE-ε4 carriers had an increased burden of β-amyloid in cortical regions and progressive accumulation of tau pathology in parietal, occipital, lateral, and medial temporal cortices than APOE-ε4 non-carriers [34]. In addition, the interaction between APOE-ε4 and higher levels of tau pathology evaluated by positron emission tomography was associated with memory impairment independently of the burden of β-amyloid in 297 participants without dementia from the Alzheimer’s Disease Neuroimaging Initiative (ADNI) database [6]. In another study, the presence of APOE-ε4 and high levels of plasma p-tau 181 was associated with poor cognitive performance among 630 older individuals without dementia from the ADNI database, independent of the burden of β-amyloid pathology [7]. Moreover, having at least one APOE-ε4 allele was also associated with a high risk of AD in 267 Catholic sisters included in the Nun Study. In addition, the severity of NP and NFT included as covariates in the regression model were also independent predictors of AD. However, similarly to our findings, cerebrovascular risk factors, such as macro and microinfarcts and atherosclerosis in the Circle of Willis, were not significant predictors of cognitive impairment [5].

One prior study using mediation analysis to evaluate the indirect pathway from which APOE alleles impact late-life cognitive impairment via cerebrovascular lesions, AD-pathology, and non-AD neuropathologies was conducted using autopsy data from 1671 individuals who had a different sociodemographic profile compared to our sample (93.2% of APOE-ε4 carriers were White and had a mean education of 16.8 years). In this study, AD-pathology also mediated the association between APOE-ε4 and late-life cognitive impairment. In addition, the association between APOE-ε4 and cognition was also mediated through LBD, TDP-43, and CAA, but only among those with clinical dementia by the time of death. Cerebrovascular lesions were not significant mediators, and the protective effects of the APOE-ε2 allele against dementia were mediated only by the AD-pathology [19].

According to these findings from different populations, the APOE allele association with cognition is probably mainly via AD-pathology [5, 17, 18, 35, 36]. APOE-ε4 enhanced the burden of oligomeric β-amyloid at the synapse, which in turn is associated with synapse shrinking and loss, a known cause of the cognitive decline in AD [33]. Furthermore, APOE-ε4 has been related to reduced β-amyloid clearance and tau spreading [34, 36]. The clearance of β-amyloid from the brain is a critical process for maintaining cognitive function, and impairment of this process has been considered one of the main links between APOE-ε4 and AD [37]. However, recent data showed that an old woman with presenilin 1 mutation (PSEN1-E280A) and APOE3 Christchurch mutation in homozygosis had only mild cognitive impairment in the presence of a high burden of β-amyloid in the brain, but a very low level of tau pathology when compared with other cases with this presenilin mutation [38]. This finding brings doubt to the hypothesis of the significance of the relationship between reduced β-amyloid clearance in AD associated with APOE-ε4.

In addition, experimental studies with animals suggest that β-amyloid and tau are associated with blood vessel dysfunction and blood–brain-barrier (BBB) damage, which could facilitate the entrance of neurotoxic plasma-derived components into the brain [39]. In humans, BBB disruption is also related to cognitive dysfunction [40]. These mechanisms could explain the association between APOE-ε4 and worse cognitive abilities via the NFT and NP.

While our findings did not reveal an indirect association between APOE alleles and cognitive abilities via non-AD-related lesions, autopsy studies suggest that the APOE-ε4 allele is associated with an increased burden of α-synuclein, which is a pathological hallmark of LBD [41, 42]. One prior study also found an association between APOE and hippocampal sclerosis through TDP-43 [17, 18]. Future studies with larger samples will be important to confirm the underlying mechanisms linking APOE to cognition through these non-AD neuropathologies.

Although APOE alleles are related to lipid metabolism, atherosclerosis, inflammation processes, and oxidative stress, prior studies have not found mediation effects of cerebrovascular lesions on the relationship of APOE alleles with cognition, which is in line with our results [5, 19, 43, 44].

Finally, we need to consider some limitations of our study. Although we applied a causal mediation approach to evaluate the indirect and direct associations of APOE alleles with cognitive abilities, we used cross-sectional data unsuitable for causal inference. Longitudinal studies will be essential to confirm these causal pathways. Moreover, the frequency of APOE-ε2 alleles, as well as each of the six APOE genotypes (ε2/ε2, ε2/ε3, ε2/ε4, ε3/ε3, ε3/ε4, ε4/ε4), was small, which preclude the use of individual genotypes in our analysis. Also, we had missing data for TDP-43, which may have brought imprecision to these estimations due to the small sample size. Furthermore, we used a dichotomous classification for LBD, TDP-43, and cerebrovascular pathologies, while the NFT and NPs were classified as ordinal variables using established staging systems. Although the binary classifications for LBD, TDP-43, and cerebrovascular pathologies were based on their strong association with cognitive abilities, the absence of mediation effects between APOE and these lesions could be due to these dichotomous classifications. In addition, although our Biobank has implemented the ABC scoring system for the neuropathologic assessment of AD [45], most cases included in this study did not have this information.

On the other hand, we used autopsy material from an admixed sample of a low-to-middle-income country with many people with low educational attainment. Moreover, the Biobank for Aging Studies is the largest in Latin America, with a community-based sample with participants with different levels of dementia severity. These characteristics allowed us to compare our findings with previous results on less diverse and higher educated populations.

## Conclusion

The association between the APOE-ε4 allele and cognitive abilities was mediated by the burden of NFT and NP, the hallmarks of AD. Non-AD neurodegenerative and cerebrovascular lesions did not mediate the pathway linking APOE to cognition. These findings shed light on pathological mechanisms that may explain the relationship between APOE-ε4, the main genetic risk factor for late-onset AD, and cognitive impairment in an ethnically diverse population and confirm previous results observed in other studies with mainly White participants. In addition, it provides evidence to support future disease-modifying drug development targeting APOE-ε4, which seems to modulate biological processes associated with key AD neuropathological markers.

### Supplementary Information

**Additional file 1: Fig 1.** Flowchart of the study participants. **Table 1.** Brain tissue staining protocol of the Biobank for Aging Studies. **Table 2.** Decomposition of the total, direct, and indirect effects through neurodegenerative and cerebrovascular lesions of the association between Apolipoprotein E gene ε4 allele (APOE-ε4) and cognitive abilities (n = 648).
