# Supplementary material for: Association between APOE-ε4 allele and cognitive function is mediated by Alzheimer’s disease pathology: a population-based autopsy study in an admixed sample
Source: Acta Neuropathol Commun. 2023 Dec 19;11:205. doi: 10.1186/s40478-023-01681-z (PMC10731799; doi:10.1186/s40478-023-01681-z)
Supplement: Supplementary file 1 — Additional file 1: Fig 1. Flowchart of the study participants. Table 1. Brain tissue staining protocol of the Biobank for Aging Studies. Table 2. Decomposition of the total, direct, and indirect effects through neurodegenerative and cerebrovascular lesions of the association between Apolipoprotein E gene ε4 allele (APOE-ε4) and cognitive abilities (n = 648). [file 40478_2023_1681_MOESM1_ESM.docx]

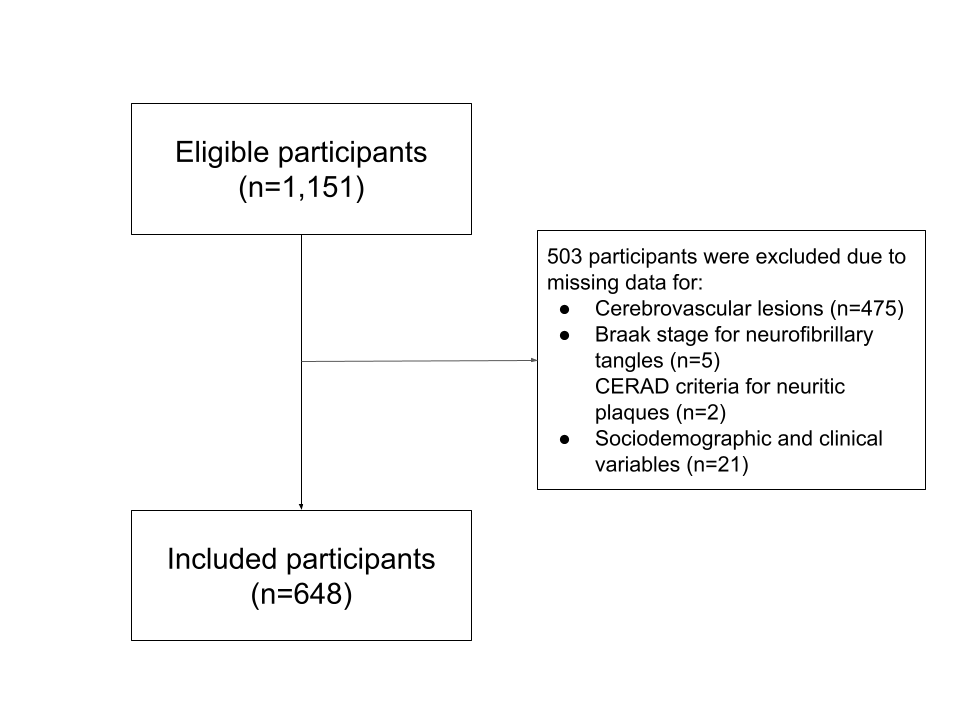


**Supplementary Figure 1.** Flowchart of the study participants.

**Supplementary Table 1.** Brain tissue staining protocol of the Biobank for Aging Studies.

| Brain region | H&E | β-amyloid | p-tau | α-synuclein | TDP-43 |
| --- | --- | --- | --- | --- | --- |
| Middle frontal gyrus | X | X | X✝ | X* | X# |
| Middle and superior temporal gyrus | X |  | X | X* | X# |
| Visual cortex | X |  | X✝ |  |  |
| Hippocampi | X | X | X | X | X |
| Amygdala | X |  | X | X | X |
| Basal ganglia | X | X☦ |  |  |  |
| Thalamus | X |  |  |  |  |
| Midbrain | X | X☦ |  | X* |  |
| Pons with locus coeruleus |  |  |  | X |  |
| Medulla oblongata |  |  |  | X* |  |
| Cerebellum |  | X☦ |  |  |  |
| H&E= hematoxylin and eosin staining; Additional staining may be ordered by the neuropathologist.  ☦=beta-amyloid-positive cases; ✝=p-tau-positive cases; alpha-synuclein-positive cases; #=TDP-43-positive cases. | | | | | |

**Supplementary Table 2.** Decomposition of the total, direct, and indirect effects through neurodegenerative and cerebrovascular lesions of the association between Apolipoprotein E gene ε4 allele (APOE-ε4) and cognitive abilities (n=648).

|  |  |  |  |  |  |  |  |  |
| --- | --- | --- | --- | --- | --- | --- | --- | --- |
| **Outcome:** cognitive abilities measured by the Clinical Dementia Rating Sum of Boxes | | | | | | | | |
|  | **Total Effect** | | **Direct effect** | | **Indirect effect** | | **Proportion Mediated** | |
|  | Estimate (95% CI) | *P* | Estimate (95% CI) | *P* | Estimate (95% CI) | *P* | Estimate (95% CI) | *P* |
| **Neurodegenerative mediators** |  |  |  |  |  |  |  |  |
| Neurofibrillary tangles (n=648) | 2.13(1.14; 3.12) | <0.001 | 1.34(0.45; 2.28) | 0.01 | 0.78(0.39; 1.22) | <0.001 | 0.37(0.20; 0.67) | <0.001 |
| Neuritic plaques (n=648) | 1.82(0.66; 3.01) | <0.001 | 0.95(-0.07; 1.96) | 0.07 | 0.87(0.39; 1.40) | <0.001 | 0.48(0.24; 1.07) | <0.001 |
| TDP-43 (n=310) | 1.16(-0.15; 2.65) | 0.08 | 1.06(-0.21; 2.42) | 0.09 | 0.10(-0.15; 0.50) | 0.42 | 0.09(-0.40; 0.61) | 0.44 |
| Lewy bodies disease (n=617) | 1.98(1.01; 3.06) | <0.001 | 1.99(1.03; 3.07) | <0.001 | -0.004(-0.11; 0.12) | 0.92 | -0.002(-0.06; 0.06) | 0.92 |
| **Cerebrovascular mediators** |  |  |  |  |  |  |  |  |
| Lacunar infarcts (n=648) | 2.09(1.04; 3.11) | <0.001 | 2.03(0.99; 3.04) | <0.001 | 0.06(-0.05; 0.22) | 0.29 | 0.03(-0.03; 0.11) | 0.29 |
| Hyaline arteriosclerosis (n=648) | 2.09(1.01; 3.13) | <0.001 | 2.16(1.10; 3.22) | <0.001 | -0.06(-0.20; 0.03) | 0.19 | -0.03(-0.13; 0.01) | 0.19 |
| Cerebral Amyloid Angiopathy (n=648) | 2.09(1.11; 3.21) | <0.001 | 1.98(0.96; 3.11) | <0.001 | 0.11(-0.07; 0.32) | 0.22 | 0.05(-0.03; 019) | 0.22 |

Mediation analysis adjusted for age, sex, race, education, hypertension, diabetes, dyslipidemia, heart disease, body mass index, smoking, alcohol use, and physical activity.

APOE groups: Participants with at least one APOE-ε4 allele vs. APOE-ε4 non-carriers (reference group).
